# Rapid Radio Map Construction via Vehicle-Based SLAM in GNSS-Denied Environments

**DOI:** 10.3390/s26165049

**Published:** 2026-08-09

**Authors:** Beomju Shin, Taehun Kim, Taikjin Lee

**Affiliations:** 1 College of Information Science, Hallym University, 1 Hallymdaehak-gil, Chunchein 24252, Gangwon-do, Republic of Korea; 2 TJ LABS, 15F, 419, Teheran-ro, Gangnam-gu, Seoul 06134, Republic of Korea; steve@tjlabscorp.com (T.K.); or taikjin@kist.re.kr (T.L.); 3 Sensor System Research Center, Korea Institute of Science and Technology, 5, Hwarang-ro 14-gil, Seongbuk-gu, Seoul 02972, Republic of Korea

**Keywords:** Fingerprinting, BLE, SLAM, Smartphone, Underground Parking lot

## Abstract

This study proposes a practical framework for rapidly generating a fingerprinting-based radio map in GNSS-denied environments such as underground parking lots. The system employs vehicle-based simultaneous localization and mapping (SLAM) to estimate trajectories using data collected from onboard smartphones and an OBD2 interface. During normal driving, Bluetooth Low Energy (BLE) signals and inertial sensor measurements are recorded to construct spatial radio maps. To address device-dependent signal characteristics, Android and iOS smartphones are used simultaneously to generate platform-specific fingerprinting maps. To improve trajectory accuracy, a heading correction algorithm compensates for gyroscope drift during straight driving segments. Loop closures are then detected using both heading sequence similarity and BLE RSSI vector correlation within a graph-based SLAM framework. The optimized trajectory is integrated with BLE signal measurements to construct a spatial radio map. Experiments conducted in a large underground parking facility accommodating approximately 800 vehicles demonstrate that the entire radio map can be generated within approximately one hour of driving. The proposed approach significantly reduces the time and labor required for conventional site surveys while maintaining spatial consistency of the radio map. These results demonstrate the feasibility of scalable BLE-based indoor positioning in large GNSS-denied environments.

## 1. Introduction

Global Navigation Satellite Systems (GNSS), such as GPS, GLONASS, and Galileo, have become fundamental technologies for outdoor vehicle localization, offering high accuracy and global coverage [1]. These systems support various navigation applications including vehicle tracking, autonomous driving, and fleet management. However, GNSS signals are significantly degraded or completely unavailable in indoor or semi-indoor environments such as underground parking lots, tunnels, or dense urban infrastructures [2]. This limitation creates a critical challenge for seamless navigation systems that must operate across both outdoor and indoor environments. Consequently, alternative positioning technologies capable of operating independently of GNSS are required to enable continuous indoor localization services [3].

Recent advances in multi-frequency smartphone GNSS, assisted-GNSS, and precise point positioning services have improved positioning performance in outdoor and partially obstructed environments [4]. Under favorable satellite visibility and reliable correction availability, these techniques can provide meter-level or better accuracy Nevertheless, their performance still depends on usable satellite observations and external correction information. They therefore do not remove the need for alternative positioning infrastructures in fully GNSS-denied environments such as underground parking facilities.

Among the various alternatives to GNSS, signal fingerprinting has attracted considerable attention because of its robustness in complex indoor environments. It estimates a user’s position by comparing observed received signal strength indicator (RSSI) patterns from Wi-Fi access points, Bluetooth Low Energy (BLE) beacons, or cellular transmitters with a preconstructed radio map [5,6,7,8]. However, its performance strongly depends on the spatial accuracy and density of the radio map [9].

Conventional radio maps are typically constructed through manual site surveys, which are labor-intensive, time-consuming, and difficult to scale in large environments such as underground parking facilities. Environmental changes may also require repeated surveys, further increasing maintenance costs. Although crowdsourcing, signal simulation, and robot-assisted data collection have been investigated to reduce this burden [10,11,12,13], practical challenges remain in terms of scalability, deployment complexity, and reliability.

To address these challenges, this study proposes a vehicle-based radio map construction framework using simultaneous localization and mapping (SLAM). The proposed system collects Bluetooth Low Energy (BLE) signals and inertial sensor measurements using smartphones mounted inside a vehicle, while vehicle speed information is obtained from an OBD2 interface. By leveraging natural vehicle motion during normal driving, the system enables automated large-scale data collection without dedicated surveying equipment. To improve trajectory accuracy, a heading correction algorithm is introduced to mitigate gyroscope drift during straight driving segments. Furthermore, loop closures are detected using both heading sequence similarity and BLE RSSI vector correlation, enabling robust trajectory optimization within a graph-based SLAM framework. The optimized trajectory is then integrated with BLE signal measurements to generate a spatial radio map aligned with the physical environment.

The proposed framework is validated in a large underground parking facility accommodating approximately 800 vehicles. Experimental results demonstrate that a dense radio map can be generated within approximately one hour of driving, significantly reducing the time and labor required for conventional site surveys. These results highlight the feasibility of scalable radio map generation for BLE-based indoor positioning in GNSS-denied environments.

The main contributions of this study are summarized as follows:

- A vehicle-based SLAM framework for automated radio map construction in GNSS-denied indoor environments is proposed.
- A trajectory refinement approach combining heading correction and RSSI–heading correlation-based loop closure detection is introduced.
- A scalable radio map generation method is presented and validated in a large underground parking facility containing approximately 800 parking spaces.

The remainder of this paper is organized as follows. Section 2 reviews related work on indoor localization and automated radio map construction. Section 3 describes the proposed system architecture and data collection methodology. Section 4 presents experimental results obtained in a large underground parking environment. Finally, Section 5 concludes the paper and discusses future research directions.

## 2. Related Works

Indoor localization has been widely studied as an alternative to GNSS, particularly in environments where satellite signals are blocked or severely attenuated. Among various approaches, fingerprinting-based localization has demonstrated strong performance in complex indoor environments because it exploits signal characteristics rather than geometric or time-of-flight measurements [14,15,16]. In fingerprinting systems, a radio map is constructed during an offline phase by collecting signal measurements at known locations. During the online phase, the user’s signal observations are compared with the radio map to estimate position.

Despite its effectiveness, the performance of fingerprinting-based localization systems heavily depends on the quality and density of the radio map. The conventional method for radio map construction relies on manual site surveys, in which a human operator collects RSSI measurements using a smartphone or dedicated device while traversing the environment. Although this approach allows precise control over reference point placement and measurement quality, it is highly labor-intensive, time-consuming, and difficult to scale in large environments such as underground parking facilities [17].

To reduce the cost of manual surveys, crowdsourcing-based radio map construction methods have been proposed [18,19,20,21]. These approaches collect RSSI measurements opportunistically from smartphones carried by users during natural movement. While crowdsourcing can significantly reduce deployment cost, the absence of reliable ground-truth positions often results in sparse and noisy datasets, which may degrade localization performance. In addition, issues related to user privacy and device heterogeneity complicate the practical deployment of such systems.

Another line of research focuses on automated radio map construction using autonomous robots or dedicated mapping platforms [22,23]. These systems typically combine wheel odometry, LiDAR, and inertial sensors to estimate accurate trajectories while simultaneously collecting RSSI measurements. Although robotic platforms can provide high-quality trajectory estimates and well-structured datasets, the deployment and operational cost of such systems can limit their applicability in many real-world environments.

More recently, several studies have explored SLAM-based data collection approaches using smartphones to reduce deployment cost while maintaining trajectory estimation accuracy [24,25,26,27]. These methods utilize inertial sensors or visual–inertial odometry to reconstruct user trajectories during signal scanning. However, visual SLAM techniques often rely on stable visual features and are sensitive to illumination conditions, which can limit their performance in visually repetitive environments such as underground parking facilities. Furthermore, existing studies rarely address practical challenges related to multi-device data fusion or cross-platform signal variability.

Accurate estimation of the heading of a robot or vehicle is essential for improving the accuracy and robustness of SLAM. Gyroscopes and magnetometers are commonly used for heading estimation. However, gyroscope-based heading estimates are subject to cumulative drift over time due to sensor bias and measurement noise. In contrast, magnetometers are highly sensitive to local magnetic disturbances caused by metallic structures, electronic devices, and vehicles, which may result in unreliable heading estimates. To mitigate these limitations, various approaches have been investigated, including the use of external absolute position and velocity information provided by GNSS [28], multi-sensor fusion [29], environmental constraints, and deep-learning-based error compensation methods [30].

Overall, although significant progress has been made toward automated radio map construction, existing approaches often involve trade-offs among scalability, trajectory accuracy, and deployment cost. To address these limitations, this study proposes a vehicle-based SLAM framework that integrates BLE fingerprinting, trajectory refinement, and cross-platform data collection to enable efficient and scalable radio map generation in large GNSS-denied environments.

## 3. Proposed Systems

In this study, we address the need for alternative navigation solutions in environments such as underground parking facilities, where GNSS signals are unavailable. In such settings, positioning must rely on alternative signals, including BLE, and is typically implemented through fingerprinting-based approaches. The key requirement for these systems is the efficient construction of a radio map that represents the spatial distribution of signal characteristics. However, conventional radio map generation often relies on labor-intensive site surveys, which limits scalability in large indoor environments.

To overcome this limitation, we focus on rapidly constructing a fingerprinting-based radio map using SLAM. The proposed method leverages BLE signals together with vehicle-based sensor measurements to enable efficient data collection in GNSS-denied environments. By utilizing data acquired during natural vehicle motion, the system facilitates practical radio map generation while supporting seamless navigation between indoor and outdoor environments.

**Figure 1:**
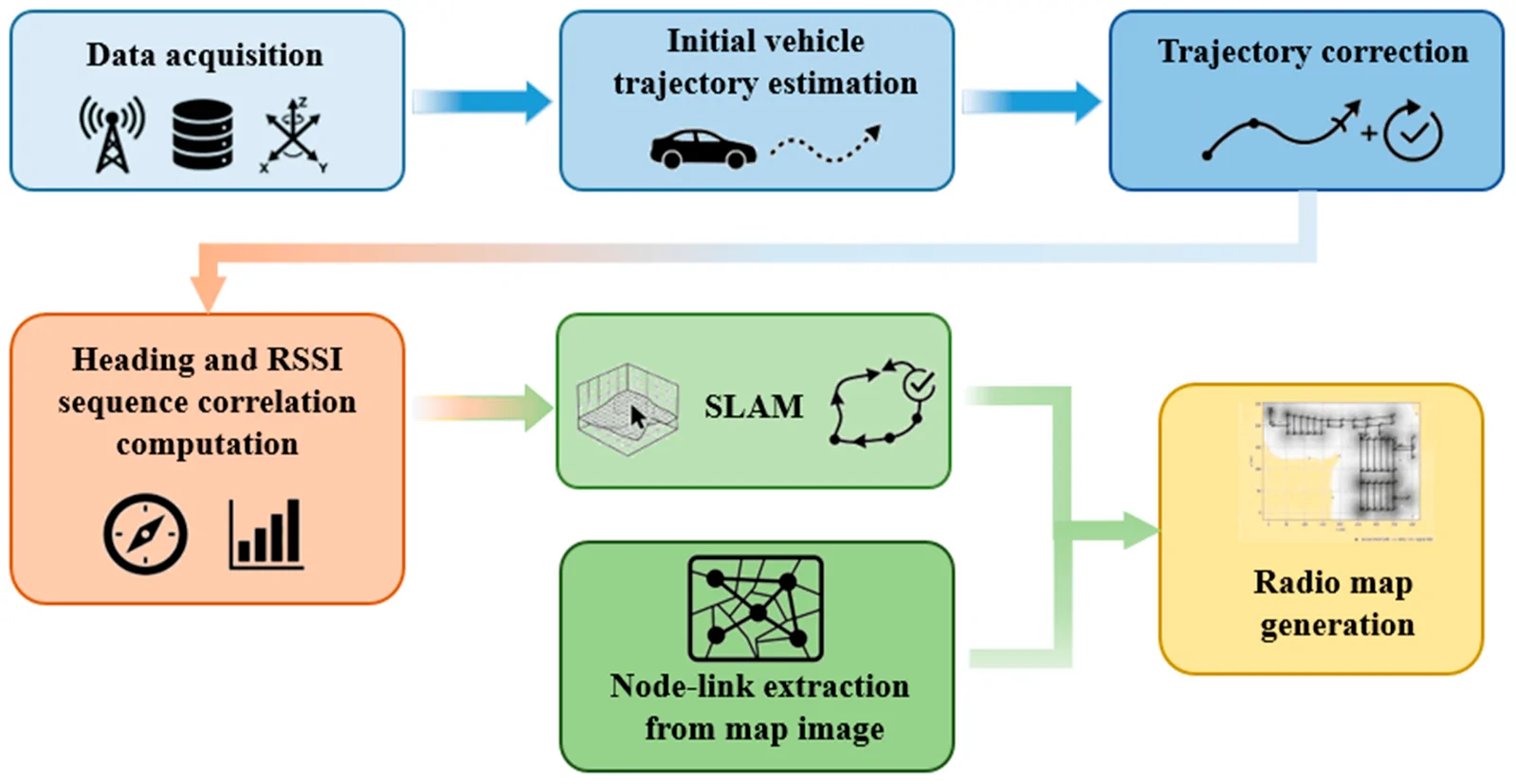
Workflow of the proposed system for automatic radio map construction.

The overall structure of the proposed system is illustrated in Figure 1. The framework consists of four main stages, each described in detail in Section 3.1, Section 3.2, Section 3.3 and Section 3.4. Section 3.1 presents the data acquisition process, in which vehicle-mounted smartphones and an OBD2 module collect inertial and signal measurements during natural driving. Section 3.2 describes the initial vehicle trajectory estimation based on dead reckoning, followed by heading correction to mitigate accumulated orientation drift. Section 3.3 introduces a correlation-based SLAM framework that integrates directional similarity with BLE signal pattern matching to support loop closure detection and improve trajectory consistency. Finally, Section 3.4 describes the extraction of node–link structures from the estimated map and the construction of the final fingerprinting radio map using BLE RSSI and SSID measurements.

### 3.1. Data Acquisition

**Figure 2:**
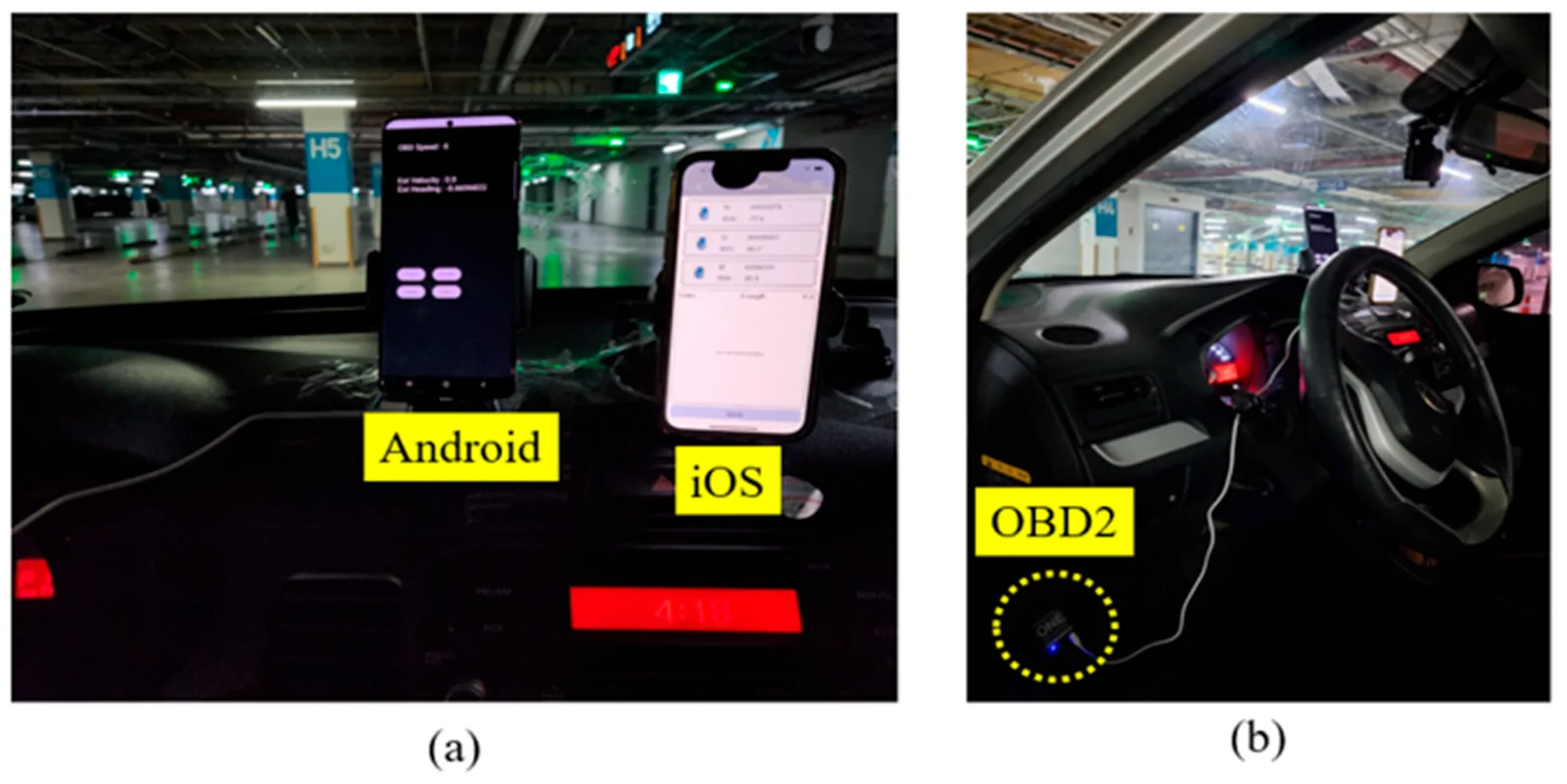
Data acquisition setup inside the vehicle. (**a**) Android and iOS smartphones mounted on the dashboard for simultaneous sensing. (**b**) OBD2 adapter connected to the Android device.

The proposed system employs two smartphones—one Android and one iOS—mounted side by side on the vehicle dashboard, as illustrated in Figure 2a. Both devices simultaneously record inertial sensor measurements, including accelerometer, gyroscope, magnetometer, orientation, and barometric pressure, as well as BLE signals consisting of RSSI values and SSIDs of nearby beacons during vehicle operation.

The Android device is additionally connected to an OBD2 adapter (Figure 2b), which is inserted into the vehicle’s diagnostic port and provides vehicle speed measurements at 40 Hz. Although OBD2 data are accessible only through the Android device, the side-by-side mounting configuration ensures that both smartphones experience identical vehicle motion. Therefore, the vehicle speed obtained from the Android device is used as a common ground-truth reference for both platforms. To synchronize the collected datasets, timestamp-based alignment is applied under the assumption that clock drift between the devices is negligible over the short data collection intervals. Due to differences in hardware design, antenna placement, and operating system–level BLE scanning policies, Android and iOS devices exhibit distinct RSSI characteristics even under identical environmental conditions. To preserve these platform-specific signal characteristics and ensure reliable localization performance, separate fingerprinting-based radio maps are constructed for each platform. Furthermore, to improve signal stability and reduce the influence of transient noise and temporal fluctuations, each driving path is traversed multiple times, and the averaged RSSI values are used for radio map construction.

### 3.2. Trajectory Estimation and Correction

Vehicle trajectory is estimated using the heading derived from the smartphone gyroscope and vehicle speed obtained from the OBD2 interface, sampled at 40 Hz. Although OBD2 data are available only on the Android device, the speed measurements are applied to both Android and iOS datasets because the two smartphones are rigidly mounted side by side on the vehicle dashboard, ensuring identical motion.

To express the angular velocity in the navigation frame, the gyroscope measurement in the body frame, $w^{b}$, is transformed using the direction cosine matrix (DCM) $c_{b}^{n}$ as shown in Equation (1):

(1) $$w^{n}=c_{b}^{n}\cdot w^{b}$$

The matrix $C_{b}^{n}$ is obtained from the smartphone’s built-in orientation estimation algorithm, which fuses accelerometer, gyroscope, and magnetometer measurements. The heading angle is then computed by integrating the yaw-rate component of $w_{z}^{n}$:

(2) $$\psi_{n}=\psi_{n-1}+w_{z}^{n}\cdot \Delta t$$

where $w_{z}^{n}$ denotes the yaw-rate component of the angular velocity in the navigation frame and Δt represents the sampling time interval, set to 0.025 s.

Since gyroscope bias causes gradual drift in the integrated heading, a correction procedure is applied during straight-driving segments. These segments are detected using a sliding-window approach in which the variance of the uncorrected heading sequence is continuously monitored. When the heading remains sufficiently stable—i.e., when the variance of the heading values within a window of size *k* falls below a predefined threshold τ—the corresponding segment is classified as straight-line motion. Under this condition, all heading values within the window are replaced by the starting heading of that segment. Otherwise, the original uncorrected headings are retained. This heading drift correction is applied according to the following rule:

(3) $${\hat{\psi }}_{i}=\left\{\begin{array}{c} \psi_{n-k},if\;Var\left(\psi_{n-k:n}\right)<\tau \\ \psi_{i},othrewise \end{array}\right.for\;i\in \left[n-k,n\right]$$

**Figure 3:**
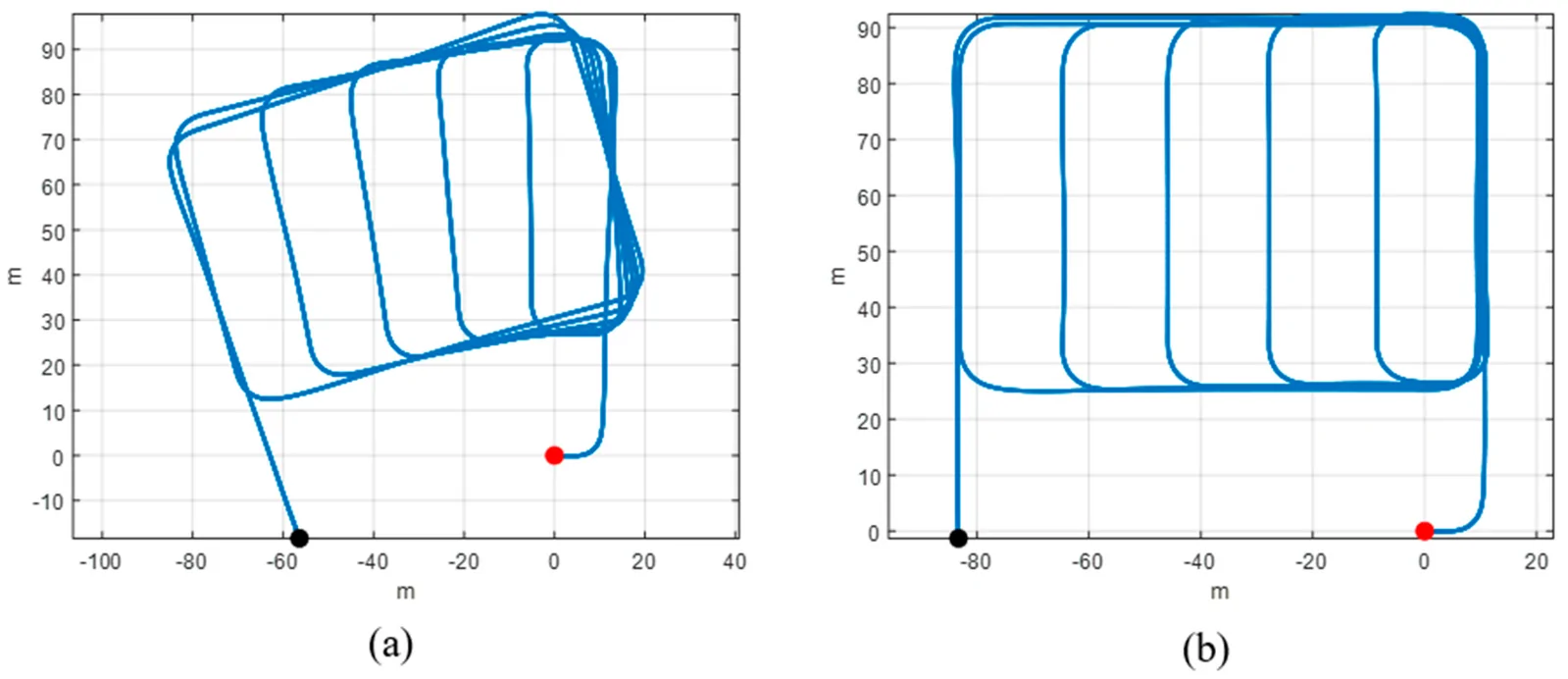
Comparison of estimated vehicle trajectories before and after heading correction. (**a**) Trajectory using uncorrected heading. (**b**) Trajectory after heading correction.

In Equation (3), the $\psi_{n-k:n}$ represents the uncorrected heading sequence from time step *n−k* to *n*. If the variance of this sequence is smaller than the threshold τ, the segment is regarded as straight-line motion. In this case, all heading values within the window are corrected by referencing the heading at time step n−k, thereby suppressing the accumulated drift. Once the heading correction is applied, the final vehicle trajectory is obtained by integrating the corrected heading and vehicle speed over time. The two-dimensional position ${\bar{x}}_{n}={\left[x_{n},y_{n}\right]}^{T}$ at time step *n* is computed as:

(4) $$\begin{array}{c} x_{n}=x_{n-1}+v_{n}\cdot \Delta t\cdot cos\left({\hat{\psi }}_{n}\right)\\ y_{n}=y_{n-1}+v_{n}\cdot \Delta t\cdot sin\left({\hat{\psi }}_{n}\right) \end{array}$$

where $v_{n}$ is the vehicle speed obtained from the OBD2 sensor. Figure 3a illustrates the trajectory generated using the uncorrected heading ψ, which exhibits significant drift due to accumulated gyroscope bias. In contrast, Figure 3b shows the trajectory obtained after applying the corrected heading $\hat{\psi }$, resulting in a more accurate and stable path estimation, particularly during straight-driving segments.

### 3.3. Correlation-Based Loop Closure and Graph SLAM Optimization

The core idea of SLAM is to estimate the trajectory of a moving platform using dead reckoning (DR), and to eliminate accumulated drift through loop closure correction. In this study, we adopt a graph-based SLAM framework, where the trajectory is represented as a set of nodes connected by links [31]. Each node corresponds to a vehicle pose and is created at regular 1-m intervals along the estimated path. The links represent motion constraints derived from sensor data and loop closure observations. Graph SLAM aims to find the optimal configuration of nodes that minimizes the total error across all constraints. To detect loop closures, this study utilizes BLE RSSI sequences in conjunction with heading information derived from inertial sensors. Given the instability of BLE signals caused by environmental noise and multipath effects, RSSI-based similarity alone may lead to incorrect loop closure detections. To improve robustness, both RSSI and heading correlations are computed as:

(5) $$\Delta {\hat{\psi }}_{k}=\frac{1}{N}\sum_{i=1}^{N}\left|{\hat{\psi }}_{k+N-i}-{\hat{\psi }}_{i}^{s}\right|$$

(6) $$\rho_{k}^{\psi }=exp\left(-\alpha \cdot \Delta {\hat{\psi }}_{k}\right)$$

(7) $$\Delta \phi_{k}=\frac{1}{N}\sum_{i=1}^{N}\sum_{j=1}^{M}\left|\phi_{k+N-i,j}-\phi_{i,j}^{s}\right|$$

(8) $$\rho_{k}^{\phi }=exp\left(-\beta \cdot \Delta \phi_{k}\right)$$

In Equation (5) and (7), *N* denotes the window size used for computing correlation scores. ${\hat{\psi }}_{i}^{s}$ represents the *i*-th heading value in the current sequence, whereas ${\hat{\psi }}_{k}$ denotes the heading value at the *k*-th node of the previously constructed trajectory. The average heading difference is computed between the current sequence and the trajectory segment spanning nodes *k-N+1* through *k*. This difference is then converted into a normalized heading correlation score, $\rho_{k}^{\psi }$, whose maximum value is 1. Similarly, for RSSI, a correlation score, $\rho_{k}^{\phi }$ is computed between the current RSSI-vector sequence and the sequence of RSSI vectors associated with nodes *k-N+1* through *k*. *M* denotes the total number of BLE beacons installed in the testbed. The scaling parameters *α* and *β* are used to normalize the heading and RSSI differences, respectively. Loop closures are identified by evaluating the heading correlation, $\rho_{k}^{\psi }$ and RSSI correlation, $\rho_{k}^{\phi }$ between the current node and previously visited nodes. A candidate is accepted as a valid loop closure only when both correlation scores exceed their respective thresholds. The threshold for the heading correlation is adaptively adjusted according to the local shape of the trajectory. A stricter threshold is applied to straight segments to prevent incorrect loop closures that could substantially distort the global SLAM graph. In straight segments, heading values are often similar across spatially unrelated locations, potentially producing artificially high correlation scores. In contrast, curved segments generally exhibit more distinctive heading patterns, allowing the threshold to be moderately relaxed without significantly compromising loop-closure accuracy. This adaptive strategy improves the robustness of loop-closure detection and helps preserve the structural consistency of the SLAM graph. Also, compared with pedestrian PDR-based RF-SLAM [24], the proposed vehicle-based system is less affected by heading drift because data collection is completed more rapidly and the smartphone is rigidly mounted. Nevertheless, residual heading errors are further reduced through loop-closure constraints in graph-SLAM optimization.

The SLAM problem is formulated as a nonlinear least-squares optimization over a set of 2D poses $X=\left\{{\bar{x}}_{1},{\bar{x}}_{2},\ldots ,{\bar{x}}_{i},\ldots {\bar{x}}_{T}\right\},$where ${\bar{x}}_{i}$ denotes the vehicle’s position at the *i*-th node. The objective is to minimize the total residuals from two types of constraints: motion constraints derived from dead reckoning and loop closure constraints based on signal similarity as:

(9) $$X^{*}=arg\underset{X}{min}T(\sum_{i=2}^{T}e_{i}^{T}\Lambda_{D}e_{i}+\sum_{j=1}^{L}e_{l_{j}}^{T}\Lambda_{L}e_{l_{j}})$$

where $X^{*}$ is the optimized trajectory, $e_{i}$ and $e_{l_{j}}$ are the residual error of DR and loop closure, respectively. $\Lambda_{D}$ and $\Lambda_{L}$ denote the information matrices associated with the DR and loop-closure constraints, respectively, representing the inverse covariance matrices of the corresponding measurement uncertainties. This formulation maintains local consistency with the DR measurements while globally correcting the trajectory using reliable loop-closure constraints. Detailed derivations of the residual terms and implementation details are provided in our previous work [24]. Ultimately, the SLAM process yields an optimized vehicle trajectory that is temporally synchronized with the corresponding RSSI vectors and heading measurements.

**Figure 4:**
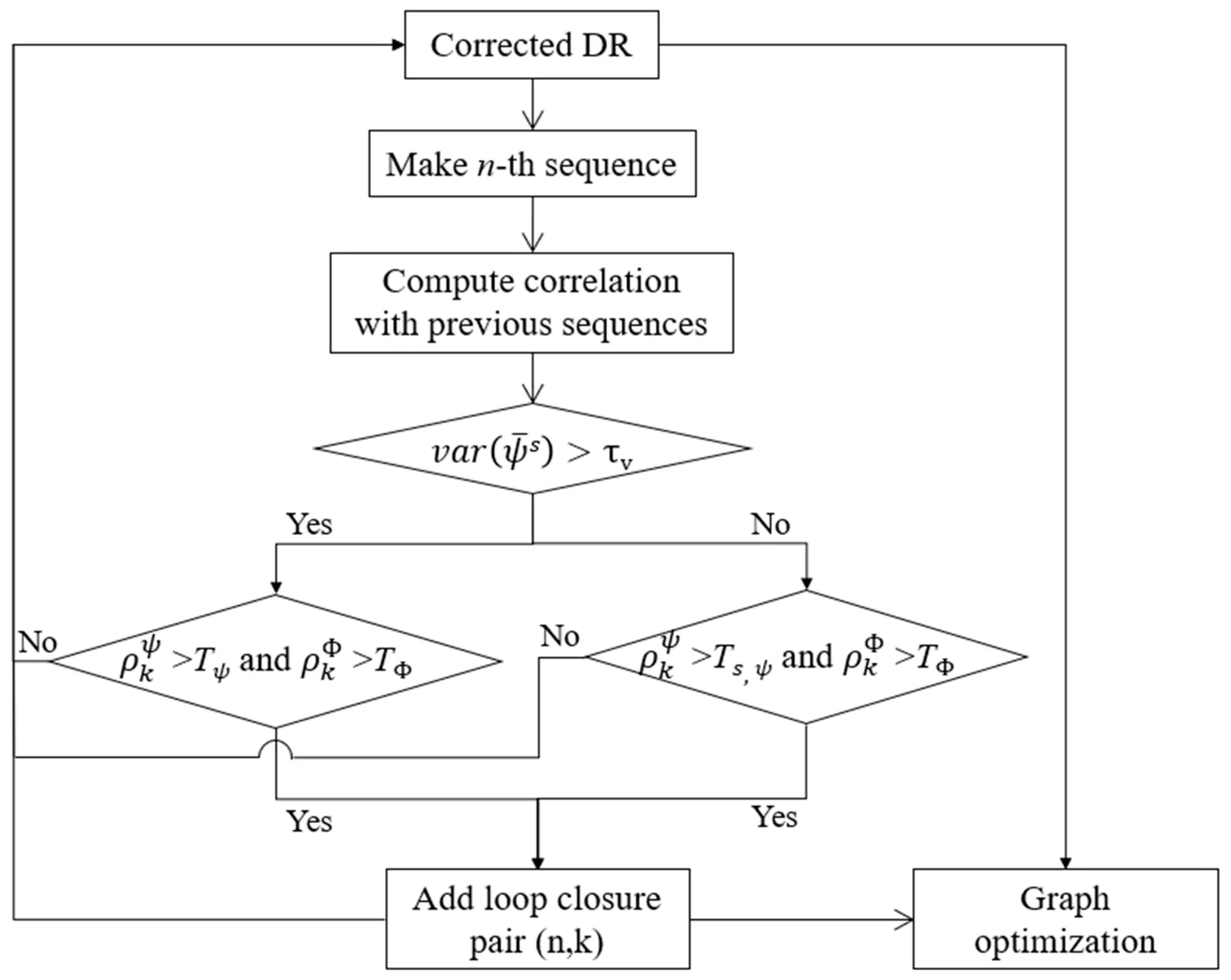
Loop closure detection process using heading and RSSI correlation.

Figure 4 shows the loop closure detection process used in the proposed SLAM system. Starting with a corrected DR trajectory, the path is divided into fixed-length sequences. For each current sequence *n*, the system computes BLE signal and heading correlations with previously visited sequences. Before evaluating the similarity, the system checks heading stability by computing the variance of the heading vector ${\bar{\psi }}^{s}$. If the variance exceeds a predefined threshold $\tau_{v}$, the sequence is likely passing through a turning or cornering section, rather than a straight path. If the segment is identified as a turning section, the system applies relaxed criteria for loop closure. In contrast, for straight, stricter thresholds are applied to prevent false positives due to the repetitive nature of straight paths. If either condition is met, the pair (*n, k*) is registered as a loop closure, and the resulting constraints are added to the SLAM graph. After all sequences are processed, graph optimization is performed to refine the trajectory.

**Figure 5:**
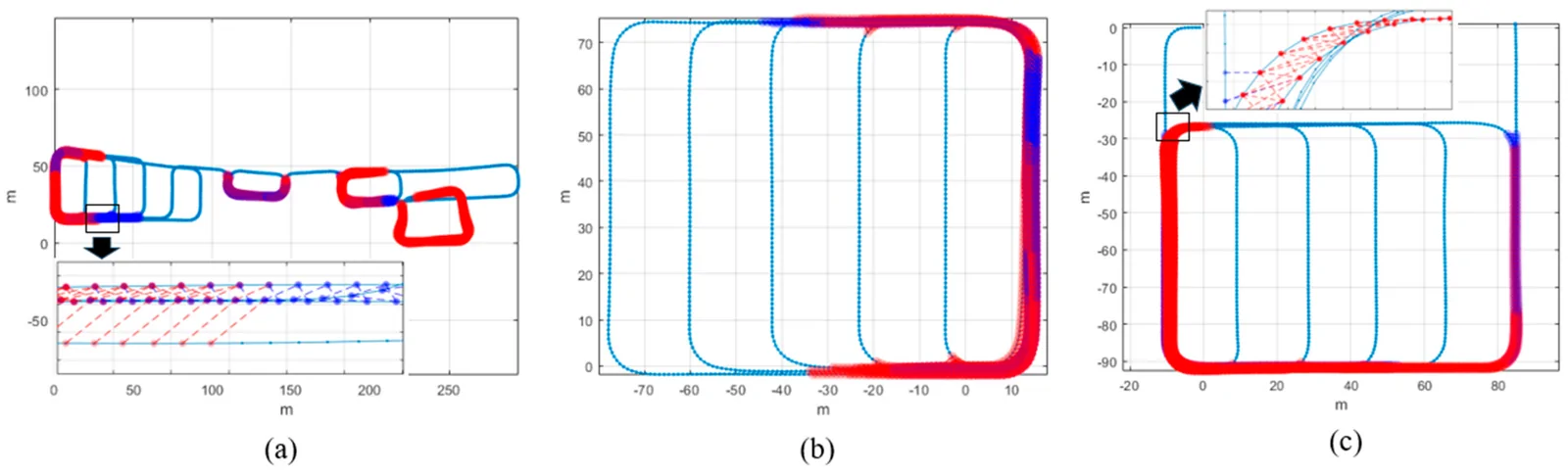
Loop closure results: red points indicate detections in non-straight segments (e.g., corners), while blue points represent those from straight segments; missed closures occur only in areas visited once. (**a**) Collection Area 1, (**b**) Collection Area 2, and (**c**) Collection Area 3.

Figure 5 illustrates the results of loop closure detection. Red points represent loop closures detected in non-straight segments (e.g., corners or turns), while blue points correspond to loop closures identified in straight segments. It is evident that the proposed method accurately detects revisited locations across various trajectory shapes. Notably, regions without loop closures are not due to detection failures but simply because those areas were traversed only once and thus lacked opportunities for loop closure detection.

### 3.4. Radio Map Construction Based on Floor Plan Alignment

**Figure 6:**
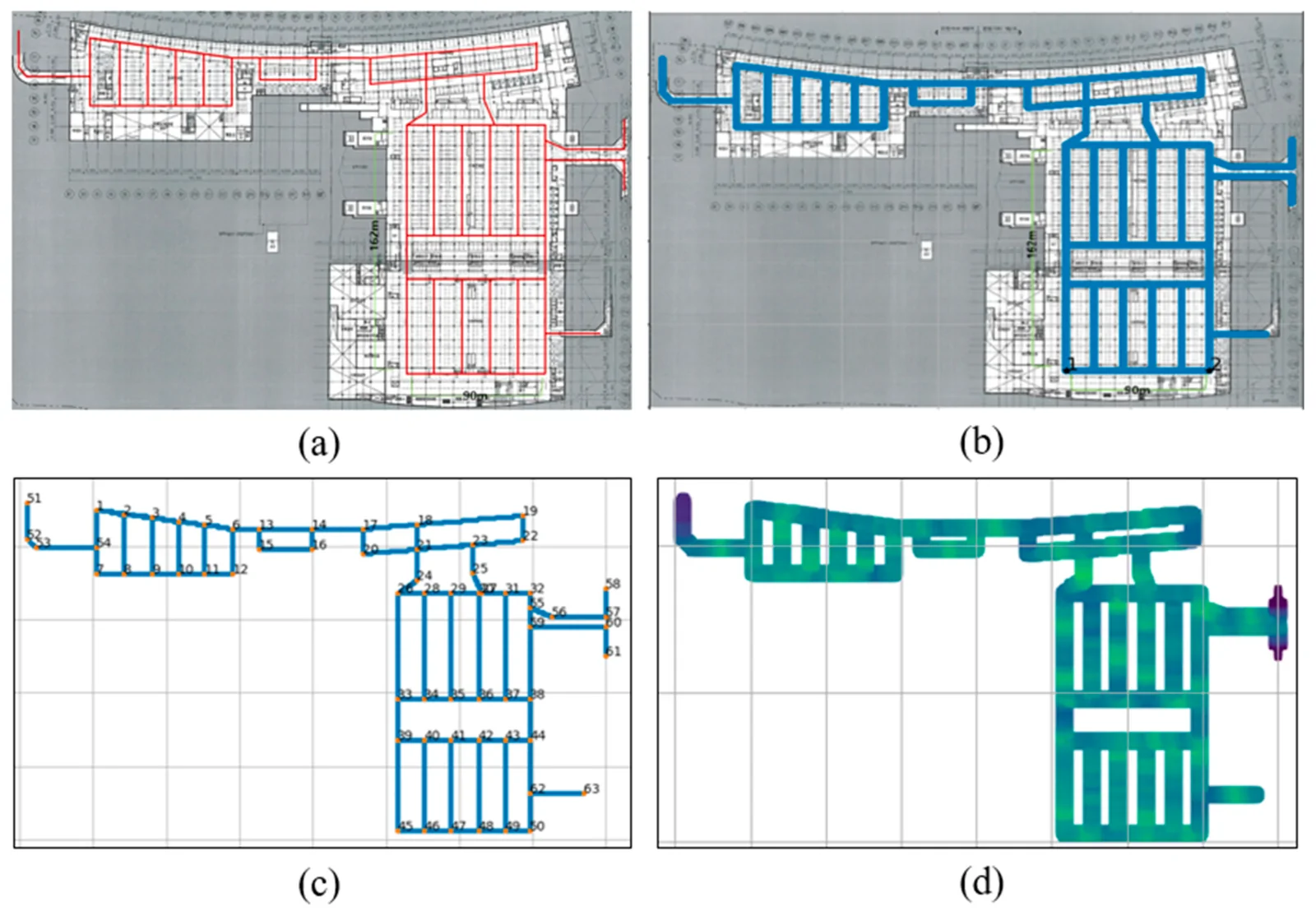
Process of constructing a floor plan-aligned radio map. (**a**) Manually drawn node-link structure on the original floor plan. (**b**) Pixel coordinates extracted from the annotated image using image processing. (**c**) Conversion of pixel coordinates into metric space and generation of node-link graph. (**d**) Alignment of the SLAM trajectory to the map nodes, followed by RSSI propagation across the road width to reflect the full drivable area.

The final output of the SLAM process is an optimized vehicle trajectory in which each node contains the associated RSSI vector and the estimated heading. Although the trajectory is optimized using sensor constraints and loop closures, residual errors such as spatial misalignment and scale distortion may still remain. To address these issues, the actual floor plan of the environment is utilized to correct the trajectory geometry. First, the drivable path structure is extracted from the floor plan by manually drawing paths along accessible vehicle routes. The resulting node-link structure is digitized by extracting pixel coordinates of the drawn paths using image processing techniques. The extracted pixel coordinates are then converted into metric coordinates using the real-world width and height of the floor plan as reference measurements. Through this process, a set of map nodes representing the actual drivable paths in meter scale is obtained. Next, the SLAM trajectory is spatially aligned with the extracted map nodes through manual correspondence between key nodes in the SLAM graph and their respective positions in the floor plan. Based on these correspondences, a geometric transformation is applied to scale and align the SLAM trajectory with the real-world layout. This alignment ensures that the estimated trajectory is consistent with the physical structure of the environment. Once thetrajectory alignment is completed, the RSSI vectors stored at each SLAM node are propagated along the map paths. This propagation distributes radio reference points across the entire driving path, including straight road segments where measurements may be sparse. As a result, the generated radio map reflects both the measured signal characteristics and the geometric constraints of the floor layout. Figure 6 illustrates the overall procedure described above.

## 4. Experimental Results

### 4.1. Experimental Environments

**Figure 7:**
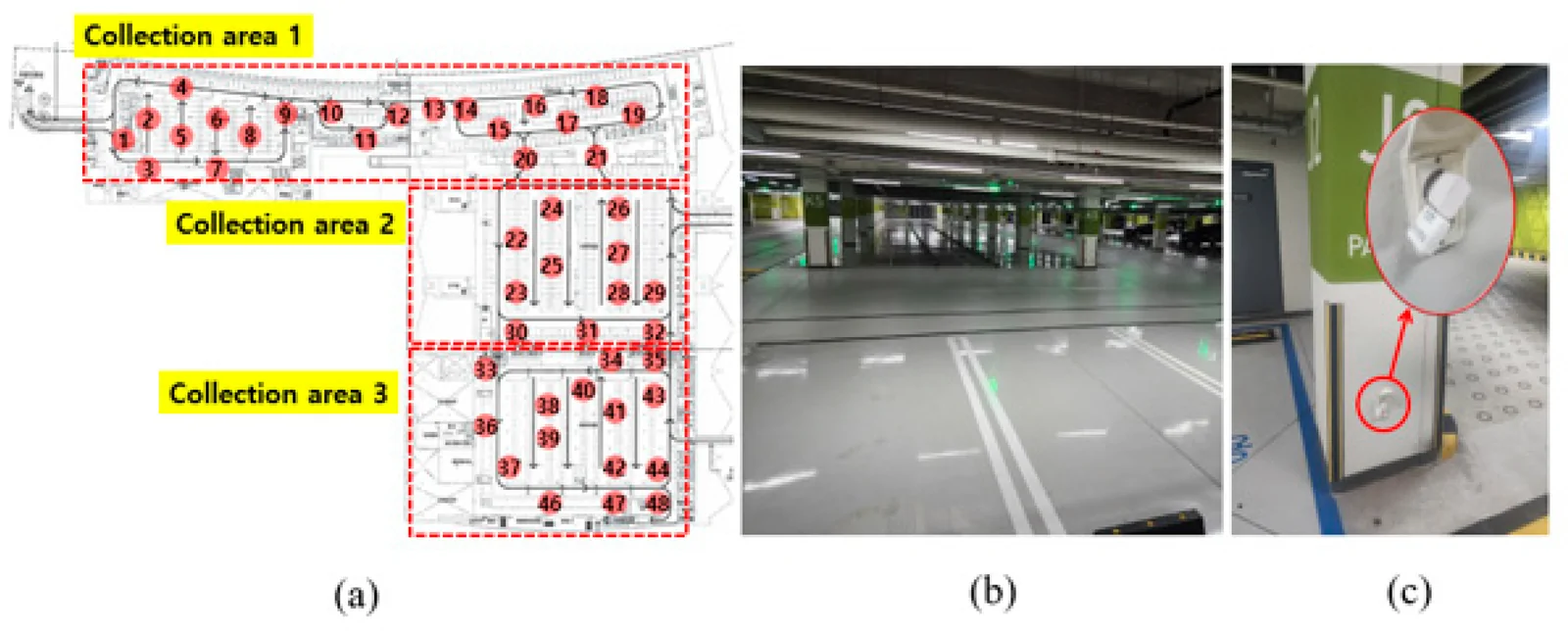
Experimental environment. (**a**) Underground parking floor plan divided into three SLAM collection areas and BLE beacon installation locations. (**b**) Photo of Collection Area 2. (**c**) Beacon plugged into a wall socket on a pillar.

To validate the proposed SLAM-assisted radio map construction framework, data collection experiments were conducted in the underground parking lot of the Songdo Convensia Convention Center located in Incheon, South Korea. As shown in Figure 7a, the test environment consists of a single underground parking level with a capacity of approximately 800 vehicles. The site was selected because it provides a large vehicle-accessible indoor space with a relatively regular structural layout and complete GNSS signal blockage, making it suitable for evaluating indoor navigation systems.

Figure 7a illustrates the floor plan of the parking facility along with the spatial distribution of the deployed BLE beacons. A total of 48 custom-built BLE beacons were installed throughout the parking area to construct a fingerprinting-based radio map. The numbered markers in the figure represent the physical installation locations of the BLE beacons. Each beacon continuously broadcasts BLE signals when connected to a standard power outlet, enabling simple and scalable deployment. The beacons were mainly mounted on structural pillars using nearby electrical outlets, with a target spacing of approximately 10 m. However, due to diverse spatial layouts and the irregular distribution of power sources, the actual beacon spacing was not perfectly uniform. Therefore, beacon placement was determined based on practical installation constraints rather than specific structural landmarks such as entrances or intersections. Figure 7b shows a representative view of the underground parking environment used for data collection, illustrating typical vehicle lanes and structural pillars present in the testbed. Figure 7c presents a close-up example of a BLE beacon connected to a wall socket on a structural pillar, demonstrating the practical installation method used during the experiment. To improve computational efficiency and maintain stable SLAM graph optimization, the parking area was divided into three spatially separated data collection regions, as shown in Figure 7a. This partitioning reduces graph complexity and facilitates reliable loop closure detection and trajectory optimization within each region during the SLAM process.

### 4.2. SLAM Results

**Figure 8:**
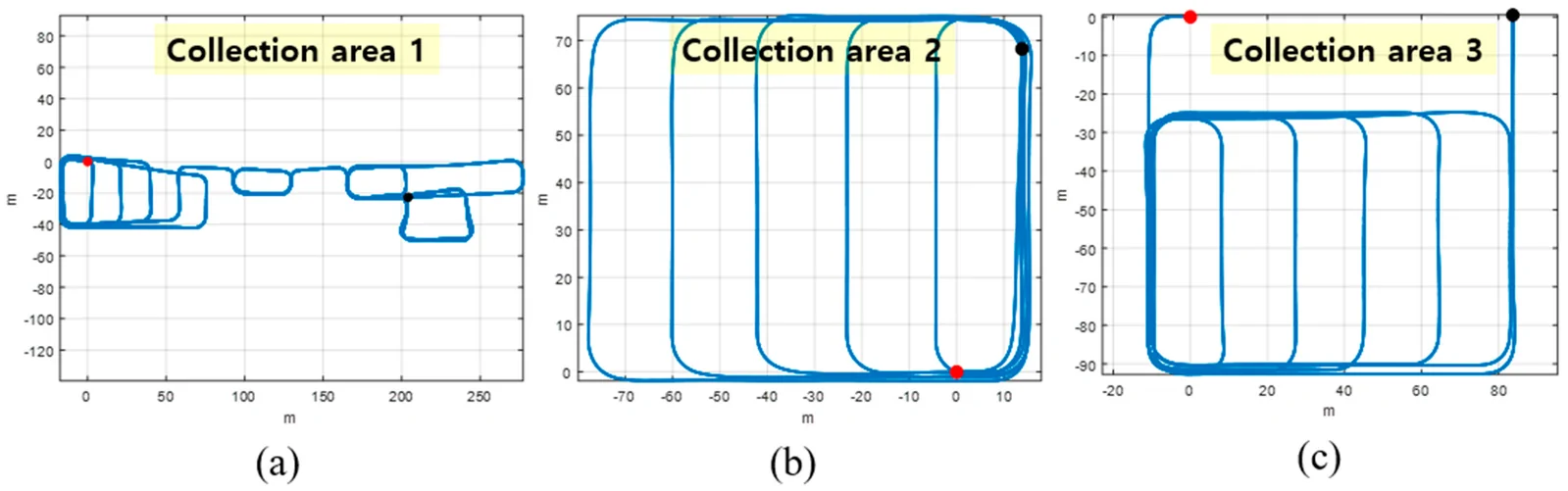
Vehicle DR trajectories with heading correction. Red and black dots indicate the start and end points, respectively. The heading-corrected DR trajectories and corresponding RSSI vector sequences serve as inputs to the SLAM process. (**a**) Collection Area 1, (**b**) Collection Area 2, and (**c**) Collection Area 3.

Before applying SLAM-based refinement, the vehicle trajectory was initially estimated using inertial sensors together with OBD-derived speed and heading correction. This preliminary trajectory provided reasonable accuracy by using OBD speed measurements for stable displacement estimation and by compensating for accumulated heading drift through motion constraints and automatic correction in straight-line segments. Figure 8 illustrates the pre-SLAM trajectories collected from three distinct regions within the test environment. In each subfigure, the red point indicates the starting position and the black point denotes the final vehicle location. Straight segments were automatically identified and exploited to reduce heading drift, resulting in notably accurate trajectory estimation along linear sections. The incorporation of OBD speed data further contributed to reliable displacement estimation. Although the overall trajectory patterns remained consistent across repeated trials, slight deviations were observed near loop regions. These discrepancies are mainly attributed to quantized OBD outputs, sensor noise, and minor environmental disturbances.

**Figure 9:**
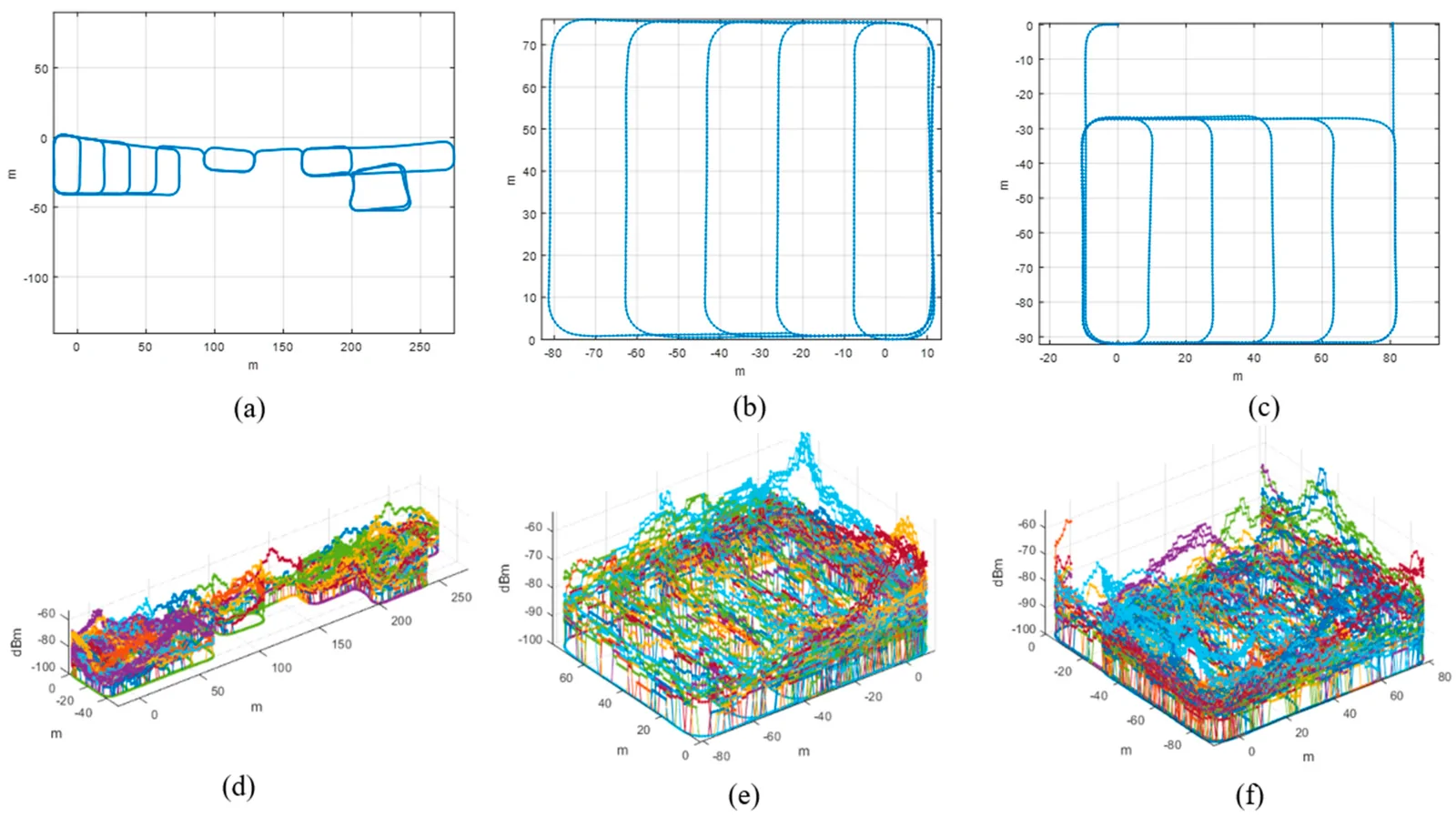
SLAM trajectories and associated RSSI vector sequences. (**a**–**c**): SLAM trajectories in Collection Areas 1, 2, and 3. (**d**–**f**): RSSI vector sequences aligned with SLAM trajectories in Collection Areas 1, 2, and 3.

Following this initial estimation, the trajectories were refined using a graph-based SLAM framework. To improve loop closure detection and enforce global consistency, both RSSI vector sequences and heading information were incorporated as complementary constraints in the optimization process. The SLAM-refined trajectories are presented in Figure 9a–c, corresponding to the three data collection regions. Compared with the pre-SLAM results, the optimized trajectories exhibit improved spatial coherence, particularly in regions involving curved motion or trajectory overlap. This enhancement in positional accuracy provides a more reliable basis for associating signal measurements with precise spatial coordinates. In addition, Figure 9d–f presents three-dimensional visualizations of RSSI vector sequences mapped along the refined trajectories, clearly illustrating the spatial correspondence between signal patterns and the SLAM-optimized paths.

**Figure 10:**
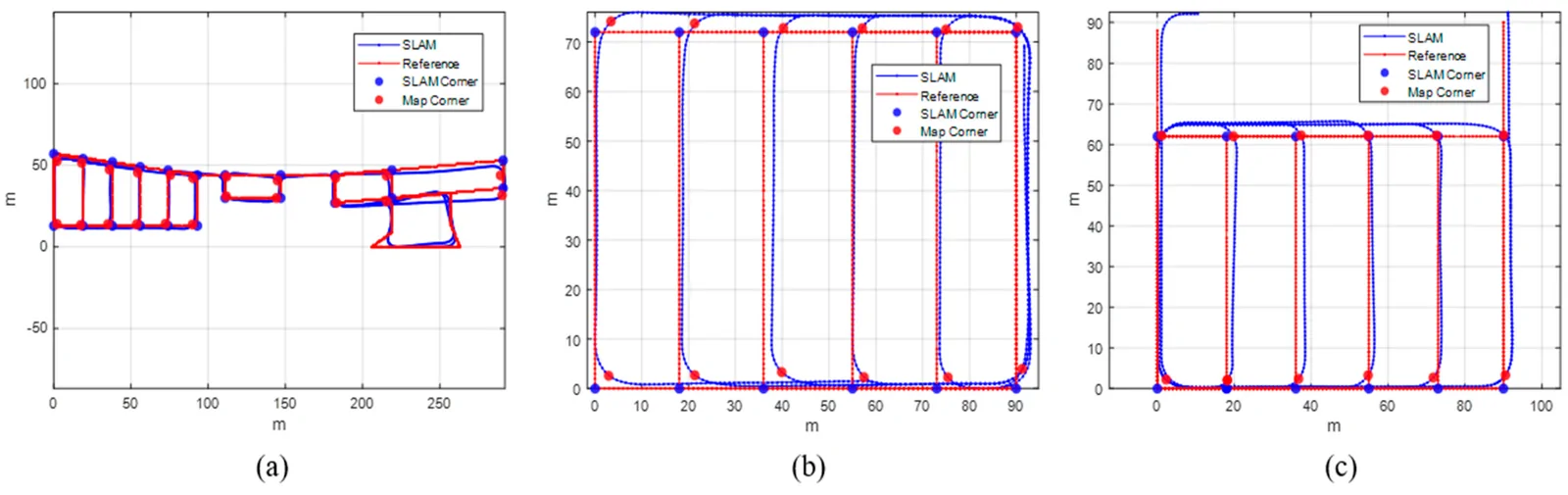
SLAM trajectory and corner-based evaluation. Red circles denote image-based corner detections, while blue dots represent corners identified via loop closure. The error is calculated as the Euclidean distance between these corresponding points. (**a**) Collection Area 1, (**b**) Collection Area 2, and (**c**) Collection Area 3.

The effectiveness of loop closure detection was evaluated by comparing the SLAM-estimated trajectory (blue line) with the reference path (red line), as shown in Figure 10. Red circles represent corner points extracted from visual features, while blue dots denote the corresponding positions identified by the proposed method through loop closure. The spatial agreement between these two sets of corner points was quantified using Euclidean distance, with key nodes extracted from both trajectories as illustrated in Figure 6. The quantitative evaluation results, expressed as RMSE for each scenario, are summarized in Table 1. These results confirm that the proposed method produces highly accurate trajectories, particularly around corner regions where loop closure plays a crucial role in correcting drift and preserving structural consistency.

### 4.3. Radio Map Construction

To construct the final radio map, the topological structure of the environment was extracted from the architectural floor plan using image processing techniques. A set of nodes and links was automatically generated to represent the physical layout as a graph. The SLAM-refined trajectory was then aligned with this node-link structure to ensure consistency with the actual spatial configuration, followed by local offset correction and scale adjustment. To better reflect the actual vehicle movement corridor, each trajectory segment was laterally expanded according to the typical road width observed in the environment. This process transformed the trajectory from a narrow line into a spatially extended region, allowing RSSI measurements to be distributed over the full drivable surface. The resulting radio map integrates the SLAM-refined trajectory, structural layout, and signal measurements, and serves as the basis for the proposed indoor positioning method, Surface Correlation (SC) [32]. This approach utilizes sequential RSSI vector patterns and compares them with the constructed radio map to enable robust localization in GNSS-denied environments.

**Figure 11:**
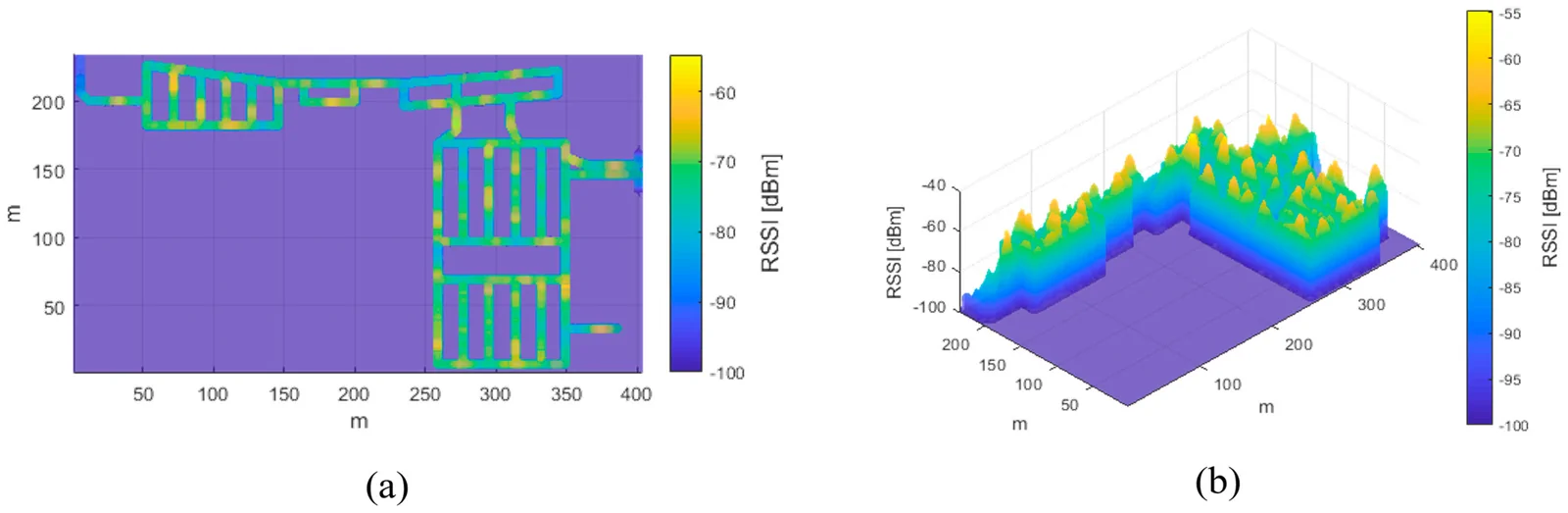
Final RSSI-based radio map constructed using SLAM-refined trajectories and RSSI vector sequences collected via an Android smartphone: (**a**) 2D (planar) view; (**b**) 3D view.

**Figure 12:**
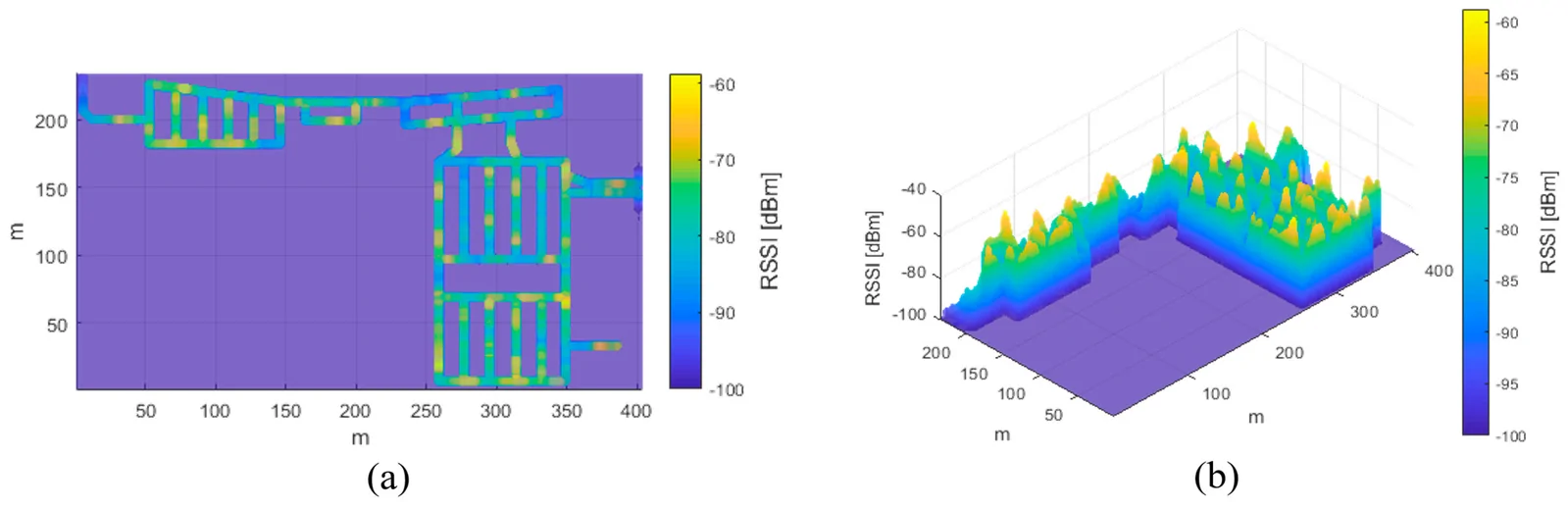
Final RSSI-based radio map constructed using SLAM-refined trajectories and RSSI vector sequences collected via an iOS smartphone: (**a**) 2D (planar) view; (**b**) 3D view.

Figure 11 and Figure 12 present the final RSSI-based radio maps constructed using sensor data collected from Android and iOS smartphones, respectively. Each figure includes both a two-dimensional planar view and a three-dimensional surface representation, illustrating the spatial distribution of RSSI measurements along the SLAM-refined trajectory. The two-dimensional views show how RSSI values are mapped across the expanded path region, while the three-dimensional views reveal the overall signal strength profile throughout the environment. The smooth continuity and spatial coherence observed in both figures demonstrate that the proposed method can generate reliable, high-coverage signal maps that are well aligned with the physical layout of the test site.

### 4.4. Discussion and Future Works

This study proposes an efficient and practical framework for automated fingerprinting-based radio map construction in GNSS-denied environments, with a particular focus on large indoor spaces such as underground parking lots. By integrating SLAM-based trajectory refinement with heading correction derived from OBD2 and inertial sensors, together with automated alignment of the trajectory to the physical floor plan using image processing, the proposed framework successfully demonstrated a scalable approach for accurate radio map generation. Unlike conventional fingerprinting methods, which require extensive manual site surveys and are therefore labor-intensive and time-consuming, the proposed system completes the entire process—from initial data collection to final radio map construction—within approximately one hour of driving, substantially improving practical deploy ability and scalability. The resulting radio maps accurately represent both the physical layout of the environment and the BLE signal distribution across drivable regions. Their effectiveness was further validated through SC-based indoor localization, confirming their suitability as robust spatial references for localization tasks.

Despite these promising results, the proposed framework has several limitations. First, although most processing steps are automated, the current system is not yet fully automatic because initial sensor installation and configuration, floor-plan preparation, and quality verification of the generated radio map still require human intervention. Second, because data are collected using a vehicle-mounted smartphone, the radio map is mainly restricted to vehicle-accessible areas and cannot directly cover pedestrian-only or narrow regions. In addition, trajectory accuracy depends on the quality of OBD2 and inertial measurements and the availability of reliable loop closures, while RSSI measurements can be affected by vehicle orientation, smartphone characteristics, surrounding vehicles, and temporal environmental changes. Since the current validation was conducted in a single underground parking facility, further experiments in diverse environments are required to verify the generalizability of the proposed method.

Future work will investigate multimodal radio map construction by integrating BLE RSSI with Wi-Fi, geomagnetic measurements. LiDAR and visual information can provide additional geometric and environmental constraints, thereby reducing trajectory uncertainty and improving map alignment when BLE or inertial measurements become unreliable. Coverage-aware path-planning methods will also be considered to achieve more uniform data collection and reduce redundant sampling. Furthermore, bidirectional data collection will be used to model and compensate for heading-dependent RSSI variations. Beyond underground parking lots, the proposed framework may be applied to warehouses, factories, logistics centers, tunnels, airport service areas, and other large GNSS-denied environments that can be traversed by vehicles, robots, or mobile carts.

## 5. Conclusions

This study proposes an efficient and scalable framework for automated fingerprinting-based radio map construction in GNSS-denied indoor environments using vehicle-mounted smartphones and an OBD2 module. By leveraging BLE signals, inertial sensor measurements, and reliable vehicle displacement information, the proposed system enables fully automated and infrastructure-independent radio map generation, thereby eliminating the need for labor-intensive manual site surveys. A key contribution of this work is the integration of graph-based SLAM trajectory refinement with tailored correction strategies, including straight-segment drift compensation and robust loop closure detection based on combined heading and RSSI correlation analysis. These techniques effectively mitigate trajectory drift, which is a major challenge in large indoor environments, and substantially improve spatial accuracy and consistency. The practicality of the proposed framework was validated through real-world experiments conducted in a large underground parking facility. The entire radio map construction pipeline was completed within approximately one hour of ordinary driving, demonstrating a substantial reduction in the time and effort required for conventional fingerprinting-based map creation. In addition, the cross-platform compatibility of the developed system further supports its applicability across diverse devices and deployment scenarios. Overall, the results confirm that the proposed framework provides an effective and practical solution for rapid radio map construction in large GNSS-denied spaces. Future work will focus on incorporating additional multi-source sensing modalities, particularly LiDAR and vision-based measurements. These sensors can provide reliable geometric and visual constraints that complement BLE, OBD2, and inertial measurements, improving trajectory estimation, floor-plan alignment, and radio map accuracy in environments with ambiguous signal patterns. Further studies will also investigate intelligent data collection, direction-aware RSSI modeling, and evaluations in warehouses, factories, tunnels, and other large GNSS-denied environments.

## Figures and Tables

**Table 1:** SLAM Performance Evaluation Results for Each Scenario.

	Correction Area 1	Correction Area 2	Correction Area 3	Total
RMSE (m)	3.73	3.62	2.20	3.35

## Data Availability

The data that support the findings of this study are available from the corresponding author upon reasonable request.
